# Mechanical, Chemical, and Processing Properties of Specimens Manufactured from Poly-Ether-Ether-Ketone (PEEK) Using 3D Printing

**DOI:** 10.3390/ma14112717

**Published:** 2021-05-21

**Authors:** Maciej Mrówka, Tomasz Machoczek, Paweł Jureczko, Kamil Joszko, Marek Gzik, Wojciech Wolański, Krzysztof Wilk

**Affiliations:** 1 Department of Theoretical and Applied Mechanics, Faculty of Mechanical Engineering, Silesian University of Technology, 18A Konarskiego Str., 44-100 Gliwice, Poland; maciej.mrowka@polsl.pl (M.M.); tomasz.machoczek@polsl.pl (T.M.); pawel.jureczko@polsl.pl (P.J.); 2 Biotechnology Center, Silesian University of Technology, Krzywoustego 8 Str., 44-100 Gliwice, Poland; 3 Department of Biomechatronics, Faculty of Biomedical Engineering, Silesian University of Technology, Roosevelta 40 Str., 41-800 Zabrze, Poland; marek.gzik@polsl.pl (M.G.); wojciech.wolanski@polsl.pl (W.W.); 4 3DGence Inc., Graniczna 66 Str., 44-178 Przyszowice, Poland; krzysztof.wilk@3dgence.com

**Keywords:** poly-ether-ether-ketone (PEEK), mechanical properties, chemical modification, 3D printing, experimental tests

## Abstract

As part of the experiments herein, the mechanical properties of specimens made of poly-ether-ether-ketone (PEEK) material using 3D printing technology were determined. Two populations of specimens were investigated, the first of which contained an amorphous structure, while the other held a crystal structure. The studies also investigated the influence of the print directionality on the mechanical properties obtained. Static tensile, three-point bending, and impact tests were carried out. The results for the effect of the structure type on the tensile properties showed that the modulus of elasticity was approximately 20% higher for the crystal than for the amorphous PEEK form. The Poisson’s ratios were similar, but the ratio was slightly higher for the amorphous samples than the crystalline ones. Furthermore, the studies included a chemical PEEK modification to increase the hydrophilicity. For this purpose, nitrite and hydroxyl groups were introduced into the chain by chemical reactions. The results demonstrate that the modified PEEK specimens had worse thermoplastic properties than the unmodified specimens.

## 1. Introduction

Modern industries, especially mechanical and biomedical engineering, are constantly searching for construction materials that may, due to enhanced useful properties and increased durability, replace the materials utilized thus far [1,2]. Continuous progress in the field of material engineering provides an opportunity to produce new or improved structural materials, which must undergo extensive research before their production can be optimized and approved for use [2,3]. The selection of material for a specific constructional application requires knowledge of its physical and chemical properties, ensuring that it will be susceptible to the required shaping, in order to obtain the finished product. Furthermore, the material should meet both economic and environmental criteria (i.e., for future recycling) [4,5,6]. Currently, a main focus of the manufacturing industry is determining the possibility of composite material applications, which often replaces those in previous use [7,8]. Polymer materials can become the matrix of the composite, which, depending on the type of filler, can change the physicochemical or biological properties that are most desirable in a given situation [9,10]. Choosing the right filler can increase mechanical strength and reduce abrasion at the same time [11,12]. An important aspect of the new materials is their non-toxicity to the environment, understood as both the impact of the entire material and that of products of possible degradation [13,14].

Poly-ether-ether-ketone (PEEK), owing to its special properties, has become a polymer that has been utilized within many industries, including the medical industry [15]. This semi-crystalline thermoplastic, belonging to the poly-ether-ether-ketone family, is characterized by a unique combination of high mechanical properties and resistance to chemicals [16,17,18]. An additional advantage of PEEK is the lack of cytotoxicity towards normal cells of the human body. Thus, it can be successfully used as a biomaterial [19,20]. Thanks to its high mechanical strength, Young’s modulus similar to that of bone, improved load distribution compared to titanium biomaterials, and lower rigidity in comparison to steel implants, PEEK has become an alternative to classical solutions [21,22,23]. Poly-ether-ether-ketone is also characterized by its high thermal stability, and due to its melting point oscillating at approximately 340 °C, PEEK is suitable for use at temperatures reaching 260 °C. With the development of additive technologies for high-temperature printing, PEEK began to be used in 3D printing of thermoplastics by liquid thermoplastic molding (fused deposition modeling, FDM) [24,25,26]. However, PEEK itself is highly hydrophobic. It promotes adhesion of bacterial cells from the environment to its surface [27]. One way to bypass the tedious and costly process of frequent sterilization is to modify the chemical structure of PEEK to give it more hydrophilic characteristics. This effect can be achieved by introducing nitro groups to the structure of the poly-ether-ether-ketone chain in different degrees of saturation, reducing the ketone group to an alcohol one, and introducing the hydroxyl group to the main chain [28].

Considering the above advantages of PEEK, especially its mechanical properties and its Young’s modulus indicating similarity to bone properties, gives high hopes for the possibility of printing dedicated implants with a better load distribution compared to steel implants. However, in order to clearly determine whether this material is suitable for 3D printing and whether it is possible to improve its biological properties, a series of experiments involving standard test methods [29,30] must be carried out. There are known studies focused on the analysis of printed PEEK samples [31,32,33]. Most of them concerned the parameters of the printing process, such as printing speed and temperature, layer thickness, and the degree of filling and laying of layers. According to these sources, it is possible to obtain optimal processing parameters ensuring the best mechanical properties. However, there is a lack of research analysing the influence of the structure and chemical parameters on the properties of the printed PEEK.

Therefore, the aim of the work was to evaluate the mechanical properties of specimens made of poly-ether-ether-ketone material using 3D printing technology. We assessed the impact of 3D printing direction and crystal or amorphous structure of the material on the mechanical properties. Furthermore, the hydrophilic nature of this material was improved by adding nitro groups with various degrees of saturation into the structure of the PEEK chain.

## 2. Materials and Methods

The material called PEEK 151G was purchased from Victrex, (Victrex, Lancashire, UK). The samples were manufactured according to the ASTM D790-02 Standard Test Method [29] with the use of incremental technology through FDM, which is a method of forming liquid thermoplastic.

The printer used to produce the samples was an INDUSTRY F340 from 3DGence (3DGence, Przyszowice, Poland). Optimal parameters of the printing process were taken according to [24]. The using printing parameters were speed of 30 mm/s, layer thickness of 0.15 mm, temperature of 425 °C, and filling ratio of 100%.

**Figure 1:**
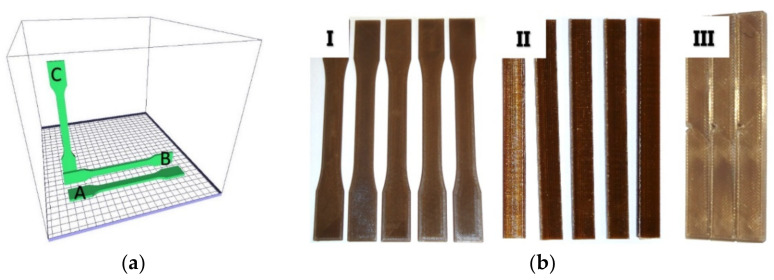
(**a**) Geometric orientation of the printed samples with the marked printing directions:(A) through thickness, (B) transverse, (C) longitudinal; (**b**) printed specimens used for: I—tensile testing, II—three-point bending testing, III—impact testing.

The sample orientation during the manufacturing process is shown in Figure 1. The specimens were created in three mutually perpendicular directions:

Type A: Specimen at the incremental manufacturing stage adjacent to the printer table with the largest surface area—through the thickness direction;

Type B: Specimen at the incremental manufacturing stage adjacent to the printer table with a smaller surface area—transverse direction;

Type C: Specimen at the incremental manufacturing stage adjacent to the printer table with the smallest surface area—longitudinal direction.

The surface of the samples after manufacturing was not modified with any chemical substances (solvents) or by any mechanical treatment. This allowed us to obtain an amorphous structure that was characteristic of the tested samples. In addition, a population of samples in crystalline form was prepared by the same manufacturing method. Due to the heat treatment consisting of heating the material in an induction furnace (Nabertherm, Lilenthal, Germany) for 120 min to 200 °C, then heating at this temperature for 180 min and free cooling under ambient conditions, the samples were brought to a crystalline form. Nitric acid (V) 65% [HNO_3_], sulfuric acid (VI) 96% [H_2_SO_4_], hydrochloric acid 35–38% [HCl], N,N-dimethylformamide [DMF], dimethyl sulfoxide [DMSO], and methanol [CH_3_OH] were purchased from ChemPur (Piekary Slaskie, Poland). Sodium hydrochloride [NaBH_4_] was purchased from Acros Organics (Saint Louis, MO, USA), technical acetone 70% was purchased from POCH™ (brand from Avantor™ Performance Materials, Gliwice, Poland), and demineralized water was obtained in the laboratory.

### 2.1. Strength Analysis Using a Static Uniaxial Tensile Method

**Figure 2:**
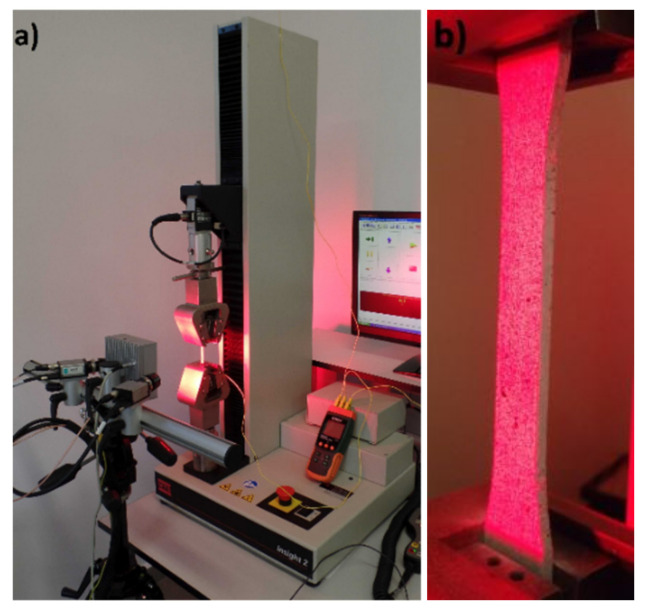
Strength test stand including (**a**) MTS Insight 2 tensile machine and DANTEC Q400 vision system; (**b**) grips used to fix a sample.

The test population consisted of 10 specimens for each printing direction. Due to the complexity of the specimen structure resulting from the manufacturing method, an experimental test was carried out using an MTS Insight 2 machine (MTS Systems, Eden Prairie, MN, USA) and a DANTEC Q400 vision system (Dantec Dynamics A/S, Skovlunde, Denmark) for non-contact deformation analysis (Figure 2). The using speed of the test was 50 mm/min. The stress at yield, elongation at yield, tensile strength at break, elongation at break, and modulus of elasticity were determined. In order to perform static tensile measurements with the use of a vision system, it was necessary to prepare a suitable background for stochastically placed markers on the measuring portion of individual samples. This action was carried out on the basis of a special preparation in aerosol, which, by definition, did not react with the material, but provided only a primer for dynamically sprayed black markers. These markers were utilized to determine the relative elongations (deformations) of the specimen in both the longitudinal and the transverse directions. Moreover, thanks to the applied vision system, Poisson’s coefficients were determined, which in the case of the analyzed, heterogeneous structures were a serious concern. The analysis was limited to only five specimens in each group, one for each direction of manufacture, as the range of forces in some cases exceeded the measuring range of the MTS Insight 2 tensile machine. The experiment was carried out at room temperature (i.e., 22 °C, humidity: 50%). As a result of the trials, a set of mechanical parameters describing the structure of the material from which the samples were made was obtained and is presented in Table 1.

### 2.2. Three-Point Bending Experiments

Three-point bending tests were carried out on specimens produced according to the ASTM D790-02 Standard Test Method [29]. The experiment was carried out on an MTS Insight 2 tensile machine. The speed utilized during the analysis was 1.65 mm/min, and the distance between the supports was 52 mm.

**Figure 3:**
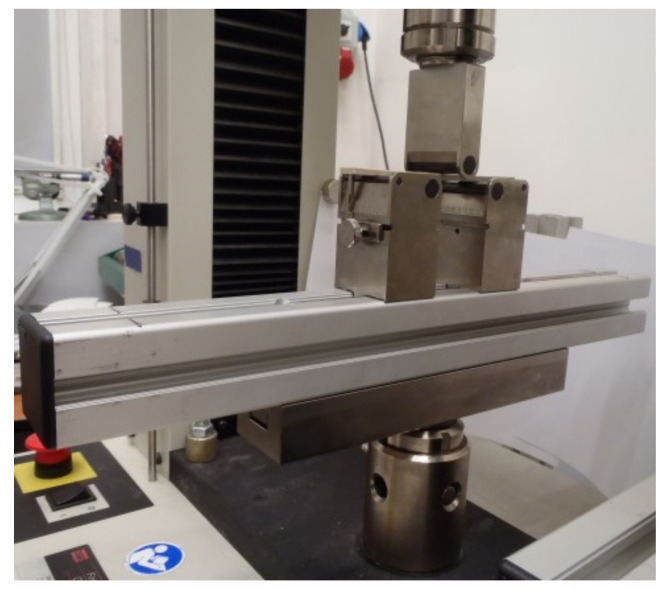
Stand for the three-point bending tests.

Each procedure was carried out at room temperature (i.e., 22 °C, humidity: 50%). The method of fixing a sample is shown in Figure 3.

### 2.3. Impact Experiments

**Figure 4:**
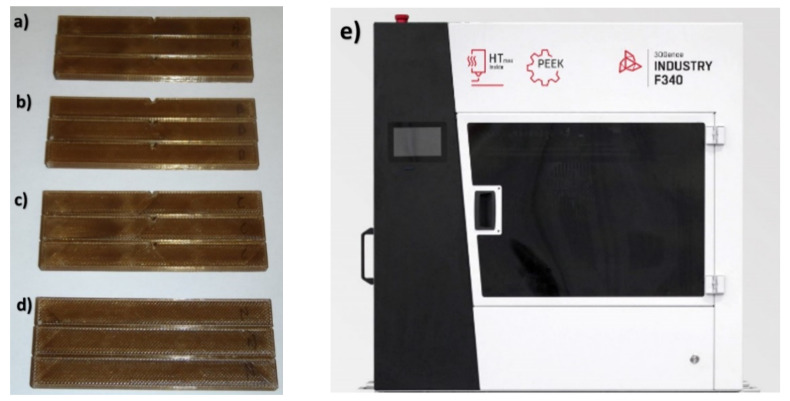
Specimens for impact testing: (**a**) Notch shape type A, (**b**) notch shape type B, (**c**) notch shape type C, (**d**) samples without notches N, (**e**) 3D printer.

Impact testing was performed on the specimens adjacent to the largest surface of the 3D printer table during the manufacturing phase. Due to the innovative nature of the issue, and due to the recommendations of the ASTM D256-02 Standard Test Method [30], the experimentation on the specimens was carried out with three different notch types (Figure 4). Additionally, specimens were tested without the recommended stress concentration component. These specimens, similar to those in the static tensile and flexural analysis, were in the form of amorphous and crystalline structures. The trials were performed using a universal impact testing machine 400/27 (VEB Werkstoffprufmaschinen, Liepzig, Germany), with a hammer weight of 0.937 kg and a hammer impact speed of 1.3048 m/s. The trials were carried out at room temperature (i.e., 22 °C, humidity: 50%).

### 2.4. Chemical Modifications of PEEK

The PEEK samples remaining after the mechanical tests were used for chemical modification. Poly-ether-ether-ketone was obtained by grinding unsuccessful prints and waste from the 3D printing process in a FRITSCH’s PULVERISETTE 19 mill machine (FRITSCH GmbH, Idar-Oberstein, Germany). As a result of grinding, a powder with a grain diameter of 1 mm was obtained. Afterwards, PEEK functionalized with nitro groups was prepared in 93% as described in the paper [28]. The reaction was carried out according to Conceiacao et al., 2008 [28]. A quantity of 30 g of ground PEEK was suspended in a mixture of 300 mL of HNO_3_ and 75 mL of H_2_SO_4_ (4:1 *v*/*v*) placed in a round-bottomed 750 mL magnetic stirrer flask (Labo24.pl, Gliwice, Poland). The flask was placed under a reflux condenser (Labo24.pl, Gliwice, Poland), on a heated magnetic stirrer (IKA, Staufen im Breisgau, Germany), and was heated at 65 °C for 60 min. Upon completion, the flask was removed and cooled, the liquid decanted, and the product, obtained in the form of precipitate, dissolved in DMF. Dissolved, modified PEEK_NO_2_ (93%) was precipitated in HCl, filtered, and then washed several times with distilled water, methanol, and acetone until the precipitate became neutral. The final product obtained was in the form of a yellow powder and was transferred to a porcelain evaporator (Labo24.pl, Gliwice, Poland) and dried for several hours in a laboratory dryer (Binder GmbH, Tuttlingen, Germany), heated to 100 °C.

The next step was the preparation of PEEK functionalized with nitro groups in 247% as described in the paper [28]. The procedure as follows was dictated by Conceiacao et al., 2008 [28]. Similar to the above procedure, 30 g of ground PEEK was suspended in a mixture of 300 mL of HNO_3_ and 75 mL of H_2_SO_4_ (4:1 *v*/*v*). The mixture was placed in a 750 mL round-bottomed flask with a magnetic stirrer. The flask was positioned under a reflux condenser, on a magnetic stirrer, and was heated at 75 °C for 90 min. After heating, the flask was removed and cooled, the liquid decanted, and the product, obtained in the form of sludge, dissolved in DMF. Dissolved, modified PEEK_NO_2_ (247%) was precipitated in HCl, filtered, and then washed several times with distilled water, methanol, and acetone until the sludge was neutralized. The final product obtained in the form of yellow powder was transferred to a porcelain evaporator and dried for several hours in a laboratory dryer heated to 100 °C.

In order to prepare PEEK_OH, the reaction was carried out according to Conceiacao et al., 2008 [28]. A quantity of 30 g of ground PEEK was added in portions, for approximately 5 min, to a 9 g NaBH_4_ solution in 750 mL DMSO placed in a 1000 mL round-bottomed flask. The flask was placed under a reflux condenser, on a magnetic stirrer, with heating and was heated at 120 °C for 180 min. After this time, the flask was removed and cooled, the liquid decanted, and the product, obtained in the form of a grey-beige powder, filtered and washed several times with distilled water, methanol, and acetone. After cleaning, the powder was transferred to a porcelain evaporator and dried for several hours in a laboratory dryer heated to 100 °C.

### 2.5. Determining Processing Properties

Approximately 10 g weights were prepared from each of the specimens obtained and placed in capsules (CORMAK, Siedlce, Poland). The capsules and their contents were placed into a KS 520/14 electro-inductive hardening furnace (Nabertherm, Lilenthal, Germany). The furnace was heated from room temperature (i.e., 20 °C) to a temperature of 450 °C at intervals of 20 °C. The weights of the modified products were checked at each level of temperature. As a result of the thermal treatment carried out in the KS 520/14 quenching furnace at a temperature of ~420°C, the waste from PEEK was plasticized in a container. FT-IR spectra were recorded using a Perkin-Elmer Spectrum Two spectrometer (Waltham, MA, USA). The examinations were carried out in the radiation range of 400–4000 cm^−1^.

## 3. Results

### 3.1. Results of the Uniaxial Tensile Testing

**Figure 5:**
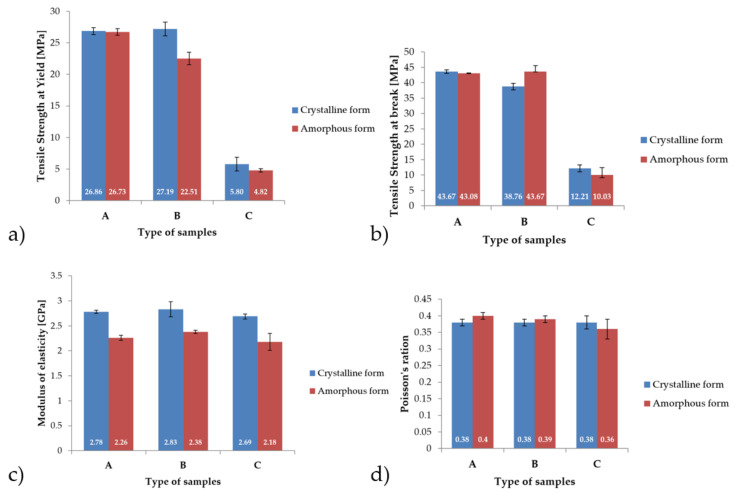
Comparison of the mechanical properties obtained during the uniaxial tensile experiments of samples type A, B and C: (**a**) Strength at Yield, (**b**) Strength at break, (**c**) Modulus of elasticity, (**d**) Poisson’s ratio.

Figure 5 presents the average values of mechanical properties for which visible changes were recorded in the uniaxial tensile testing. In the case of stress at yield, the highest mean value was recorded for samples with B-type crystal structures, with the result being 27.19 MPa. On the other hand, the lowest average value for this parameter was recorded for samples with an amorphous structure of type A, this value was 4.82 MPa. For the parameter of tensile strength at break, the highest mean value was obtained for samples of types A and B and was found to be 43.67 MPa. For the A-type sample, this number occurred in the crystal structure, and for the B-type sample, it occurred in the amorphous structure. In the case of the modulus of elasticity, it was observed that for all types of samples, i.e., A, B, and C, a higher mean value of the modulus was noted for the crystal structure, approximately 20% higher than that for the amorphous structure. The Poisson’s ratio for all samples was at a similar level and, on average, was 0.38.

**Figure 6:**
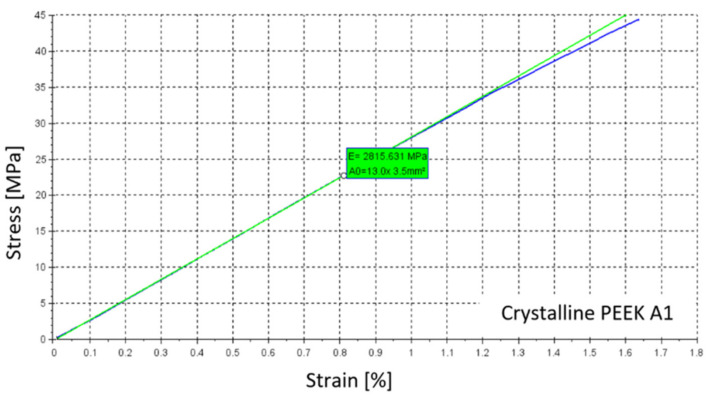
Example of the stress-strain curve of crystal poly-ether-ether-ketone specimen A1.

**Figure 7:**
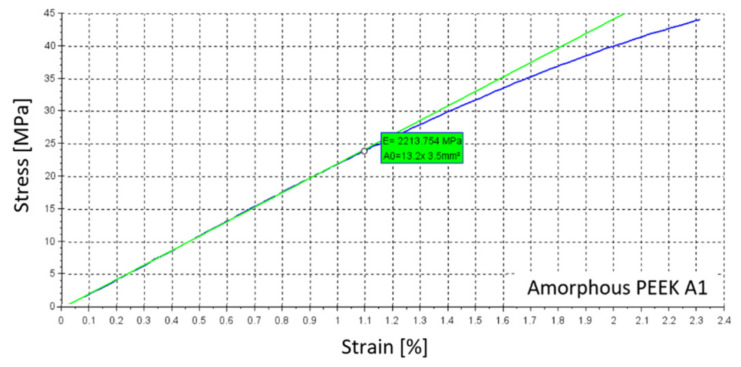
Example of the stress-strain curve of amorphous PEEK specimen A1.

Other properties, for which no significant differences were found, are summarized in Table 1 for samples with a crystalline structure. An example of the stress-strain curve which was obtained using the DANTEC Q400 vision system for specimen A1 is shown in Figure 6. In Table 2, summarized results are presented for samples with an amorphous structure, and Figure 7 shows an example of the stress-strain curve of amorphous PEEK for specimen A1.

### 3.2. Results of Three-Point Bending Testing

**Figure 8:**
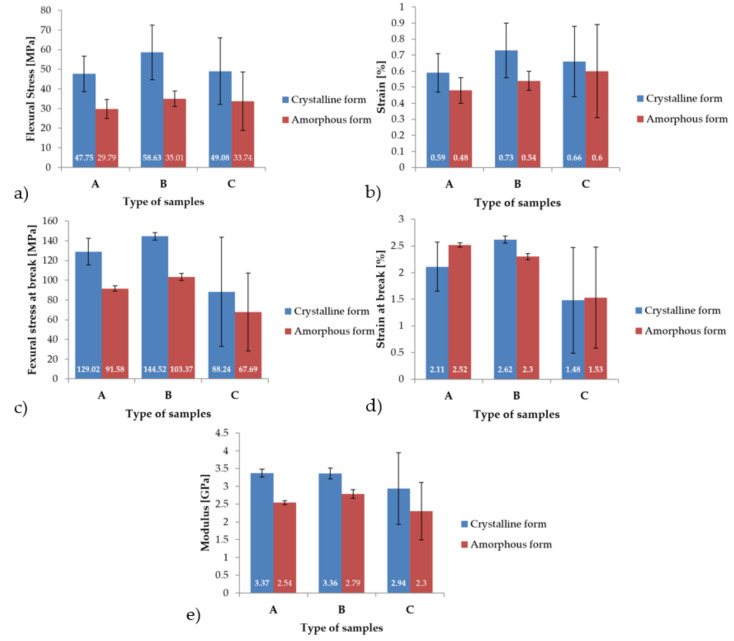
Comparison of the mechanical properties obtained during the three-point bending tests of samples types A, B and C: (**a**) Flexural stress, (**b**) Strain, (**c**) Flexural stress at break, (**d**) Strain at break, (**e**) Modulus of elasticity.

Figure 8 displays graphs containing the average values of mechanical properties recorded during the static, three-point bending tests. For the flexural stress parameter, it was observed that for all types of samples, A, B, and C, higher average values of approximately 57% were obtained for the crystal structure. The highest mean value was recorded for the B-type sample and was 58.63 MPa. The lowest mean value for the flexural stress parameter was found to be with the A sample, and it was 29.79 MPa. Furthermore, in the case of strain, the highest average values were determined to be with the crystal structure. This parameter was higher by an average of 23%. The highest mean strain, 0.73%, was recorded for sample B with a crystal structure. The lowest mean strain value was observed to be 0.48% for sample A in the amorphous phase. Additionally, for the parameter of flexural stress at break, the highest mean value was found to be 144.52 MPa for the B-type sample in the crystalline phase. The lowest mean value for flexural stress at break was recorded for the C-type sample in the amorphous phase and was 67.69 MPa. It was also observed that the modulus for all types of samples was higher for their crystal structures by an average of 27%. The highest mean value of modulus was recorded for sample A, in the crystalline phase, and was found to be 3.37 GPa. Finally, the lowest mean modulus value, 2.3 GPa, was recorded for C-type samples with an amorphous structure.

All of the mechanical properties determined during the three-point bending experiments are summarized in Table 3 and Table 4.

### 3.3. Results of Impact Testing

During the impact testing, the impact resistance of each sample was recorded, which is presented in Table 5. The highest mean value of 112.09 kJ/m^2^ was observed for the N-type sample with an amorphous structure. The smallest result was determined for a sample of type C, in the crystalline phase, and was 6.57 kJ/m^2^.

### 3.4. Infrared Spectroscopy

**Figure 9:**
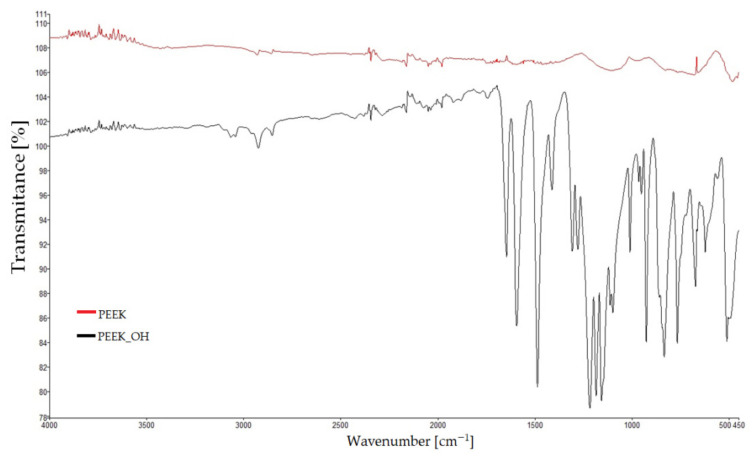
FT-IR spectrum of PEEK_OH compared to that of pure PEEK.

**Figure 10:**
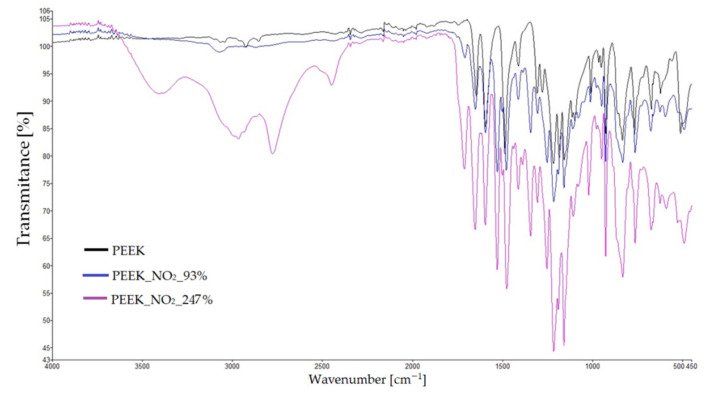
FT-IR spectra of PEEK_NO_2_—93% and PEEK_NO_2_—247% compared to that of pure PEEK.

Samples were taken in the form of a fragment of an unground filament made of PEEK, ground PEEK grains, and the three modification products. During the measurements, the spectra of unmodified PEEK were recorded, in which the carbonyl group was reduced to alcoholic (PEEK_OH) and nitrated (PEEK_NO_2_—93% and PEEK_NO_2_—247%) samples of poly-ether-ether-ketones. The results are presented in Figure 9 and Figure 10. The first figure shows the spectra of PEEK and PEEK_OH, while the second shows the spectra of PEEK, PEEK_NO_2_—93%, and PEEK_NO_2_—247%. In all spectra, the dependence of transmittance [%] on the position of the band [cm^−1^] was determined. In Figure 9, band loss in the range of 1680–1700 cm^−1^ was observed in the PEEK_OH spectrum. This band, present on the pure PEEK spectrum in the same range, comes from the carbonyl group, and its disappearance proves the reduction of the carbonyl group to a hydroxide group. Figure 10 shows the combined spectra of PEEK, PEEK_NO_2_—93%, and PEEK_NO_2_—247%. The occurrence of intense bands characteristic for nitro groups at 1540 and 1720 cm^−1^ was observed thereon. These bands do not occur in the unmodified PEEK spectrum, which proves the success of the nitriding process. Both bands are much more intense for PEEK_NO_2_—247% than for PEEK_NO_2_—93%. In all spectra, bands characteristic of aromatic compounds, differing in intensity, were also observed. Bands of aromatic compounds can be observed in the range from 1600 to 1500 cm^−1^ and at 3000 cm^−1^. In the case of the PEEK_NO_2_—247% spectrum, the band at 3000 cm^−1^ shows additionally strong intensity, coming from a high content of nitro groups in the poly-ether-ether-ketone chain.

### 3.5. Results of Chemical Modifications of PEEK

**Figure 11:**
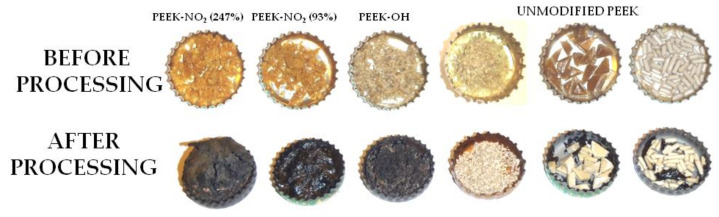
Results of thermal treatment of the obtained PEEK modification products.

In order to investigate the specific processing properties of PEEK modification products, capsules with specific weights of substances were placed in a quenching furnace and heated from 20 to 450 °C. The temperature was constantly controlled with a thermometer placed next to the furnace and was increased by 20 °C per interval. When the furnace reached the set temperature, the condition of the modification products placed in it was determined. No changes were observed in all three modified products at 80 °C, while at 100 °C, dense, eye-catching smoke escaped from the chamber when the furnace was opened, and all the samples placed in the capsules were charred. The experiment was repeated three times, but each time, the PEEK modification products charred in the temperature range of 80–100°C. Images of samples before and after the heat treatment are shown in Figure 11.

## 4. Conclusions

The resulting properties of the specimens manufactured using the 3D printing technology based on the fused deposition modeling (FDM) method showed repeatability, dependent upon the method, of their processing parameters. The effects of printing direction of layers and structural form on the mechanical properties were noted. Static uniaxial tensile testing showed that the specimens produced in direction A (i.e., with the largest surface adjacent to the printer table) had similar properties to the specimens marked as B (i.e., with the minor surface adjacent to the printer table). The lowest tensile strength was observed for specimens made in direction C (i.e., with the smallest surface adjacent to the printer table).

An analysis of literature sources [31,32,33] also showed the possibility of correcting the exemplary printing modes with the aim to improve their mechanical properties. However, the conducted research provided new information on the influence of the printing positions, structure, and chemical parameters of specimens on the properties of the printed PEEK. Thanks to the applied vision system, the Poisson’s coefficients were also determined.

The highest value of the modulus of elasticity was observed for specimens A in crystalline form during both experimental tests. The samples with a crystalline structure also had higher stiffness and, thus, increased brittleness in comparison to the amorphous samples. These tendencies were observed in the static three-point bending and uniaxial tensile tests. According to [31,32,33], our results obtained for amorphous-form samples are similar. The tensile strength had the same level of value, at about 40 MPa, but the modulus of elasticity was different due to the filling ratio. Our specimens of type A had a filling ratio of 100%, which caused a higher modulus of elasticity. The results showed that the printed PEEK exhibits exceptional tensile properties that can be achieved while maintaining a crystalline structure. This form of PEEK provides an excellent balance of mechanical properties combined with flexural and tensile characteristics.

Along with the change in the chemical structure of the modification products, the physicochemical properties of the materials changed. The resulting modification product ceased to be a thermoplastic and became a thermosetting polymer, as such, it cannot be thermally processed. While PEEK is a thermoplastic polymer, the obtained modified specimens became duromers as a result of the heat treatment.

## Figures and Tables

**Table 1:** Results of uniaxial tensile testing of the crystalline-form samples.

Name of Samples	Stress at Yield [MPa]	Strain at YIELD [mm/mm]	Stress at Break [MP]	Strain at Break [mm/mm]	Modulus of Elasticity [GPa]	Poisson’s Ratio
Sample A	26.86 ± 0.56	0.0096 ± 0	43.67 ± 0.07	0.016 ± 0	2.79 ± 0.03	0.38 ± 0
Sample B	27.19 ± 1.11	0.0096 ± 0	38.76 ± 1.9	0.013 ± 0	2.83 ± 0.15	0.38 ± 0
Sample C	5.80 ± 1.49	0.0022 ± 0	12.21 ± 2.4	0.0043 ± 0	2.69 ± 0.51	0.35 ± 0.02

**Table 2:** Results of the uniaxial tensile experiment on the amorphous-form samples.

Name of Samples	Stress at Yield [MPa]	Strain at Yield [mm/mm]	Stress at Break [MPa]	Strain at Break [mm/mm]	Modulus of Elasticity [GPa]	Poisson’s Ratio
Sample A	26.73 ± 0.52	0.011 ± 0	43.08 ± 0.12	0.022 ± 0	2.25 ± 0.05	0.40 ± 0.01
Sample B	22.51 ± 0.99	0.0094 ± 0	43.67 ± 0.13	0.021 ± 0	2.38 ± 0.03	0.39 ± 0.01
Sample C	4.82 ± 0.26	0.0022 ± 0	10.03 ± 0.87	0.0045 ± 0	2.18 ± 0.17	0.36 ± 0.02

**Table 3:** Results of three-point bending analysis of the crystalline-form samples.

Name of Samples	Stress at Yield [MPa]	Strain at Yield [%]	Stress at Break [MPa]	Strain at Break [%]	Modulus of Elasticity [GPa]
Sample A	47.75 ± 9.06	0.59 ± 0.12	129.02 ± 13.45	2.11 ± 0.46	3.37 ± 0.11
Sample B	58.63 ± 13.87	0.73 ± 0.17	144.52 ± 3.61	2.62 ± 0.07	3.36 ± 0.15
Sample C	49.08 ± 16.79	0.66 ± 0.22	88.24 ± 53.29	1.48 ± 0.99	2.94 ± 1.01

**Table 4:** Results of three-point bending tests of the amorphous-form samples.

Name of Samples	Stress at Yield [MPa]	Strain at Yield [%]	Stress at Break [MPa]	Strain at Break [%]	Modulus of Elasticity [GPa]
Sample A	29.79 ± 4.81	0.48 ± 0.08	91.58 ± 2.77	2.52 ± 0.04	2.54 ± 0.06
Sample B	35.01 ± 4.01	0.54 ± 0.06	103.37 ± 3.74	2.30 ± 0.06	2.79 ± 0.12
Sample C	33.74 ± 14.94	0.60 ±0.29	67.69 ± 39.52	1.53 ± 0.95	2.30 ± 0.81

**Table 5:** Results of impact testing for each sample.

Name of Samples	Impact Resistance [kJ/m^2^]	
	Amorphous Form	Crystalline Form
Sample A	12.80	7.66
Sample B	21.16	14.59
Sample C	12.60	6.57
Sample N	112.09	23.96

## Data Availability

Data are contained within the article.
